# Stromal immune cells expression of Siglec-15 is associated with lower T stage and better prognosis of urinary bladder cancer

**DOI:** 10.3389/fonc.2024.1437006

**Published:** 2024-12-18

**Authors:** Chengbiao Chu, Yao Fu, Jun Yang, Xiangshan Fan, Jiong Shi

**Affiliations:** Department of Pathology, Nanjing Drum Tower Hospital, Nanjing, China

**Keywords:** bladder cancer, Siglec-15, PD-L1, immunohistochemistry, clinicopathologic analyses

## Abstract

**Introduction:**

Sialic acid-binding immunoglobulin-like lectin 15 (Siglec-15) is a novel immune checkpoint, similar to programmed death-ligand (PD-L1), and has emerged as a potential target for cancer immunotherapy. Until recently, little was known about the expression and role of Siglec-15 in bladder cancer (BC).

**Methods:**

In this study, we used immunohistochemical staining to assess the expression of Siglec-15 and PD-L1 in 69 primary BC samples and analyzed their relationship with clinicopathologic characters and prognosis.

**Results:**

The expression rates of Siglec-15 in the tumor cells, stromal immune cells, and both the tumor and stromal cells were 84.1% (58/69), 50.7% (35/69), and 44.9% (31/69), respectively. The PD-L1 expression rate was 52.2% (36/69), with a positive rate of 17.4% (12/69). PD-L1 expression was inversely correlated with Siglec-15 expression, but the statistical significance was not achieved (*P* = 0.072). Low stromal Siglec-15 expression was associated with advanced tumor stage (*P* = 0.010). PD-L1 expression was associated with tumor stage (*P* = 0.008) and perineural invasion (PNI) (*P* = 0.048). Kaplan-Meier survival curves showed that stromal Siglec-15 expression was associated with a better prognosis (*P* = 0.012), although it was not an independent prognostic factor after multivariate analysis (*P* = 0.236) .

**Discussion:**

This study revealed a high expression rate of Siglec-15 in BC and may provide valuable insights for patient selection in future clinical trials.

## Introduction

Bladder cancer (BC) is the most common malignant tumor of the urinary tract, with urothelial bladder carcinoma being the most prevalent type (1). As of 2024, BC is the fourth most common cancer diagnosed in men (2). In China, the incidence rate of BC is 9.29 per 100,000 people, and this rate has been gradually increasing in recent years (3). Although bladder cancer is treated extensively with surgery, chemotherapy, and radiotherapy, the outcomes have not significantly improved over the past years (4). Muscle-invasive BC has a poor prognosis due to pelvic lymph nodes involvement or distant metastasis. Additionally, the multi-centric origin of tumors, chemotherapy resistance, and a high recurrence rate contribute to the poor disease prognosis and treatment response for BC patients (5). Therefore, there is an urgent need for new potential biomarkers and therapeutic targets in BC.

In recent years, immune checkpoint inhibitors have shown remarkable therapeutic potential for BC patients (6). Among these, Programmed Death-Ligand 1 (PD-L1) inhibitors have demonstrated good efficacy in the treatment of BC. Moreover, the PD-L1 molecules on the surface of tumor cells are closely related to the therapeutic effects and survival prognosis (7). Additionally, increasing evidence has unveiled the immunosuppressive effect of Siglec-15 and recognized it as a potential target for tumor immunotherapy. Siglec-15 is a cell surface sialic-acid-binding receptor highly conserved in vertebrates. Siglec-15 widely expressed in specific tumor cells and tumor-associated macrophages (8, 9). The regulatory role of Siglec-15 in the tumor immune response was first discovered by Wang et al. in 2019 (9). The Siglec-15 expression is frequently upregulated in certain types of cancer, including lung, colon, endometrial, bladder, renal, liver and thyroid cancers (**Supplementary Table S1**) (10, 11). The interaction between Siglec-15 and tumor-associated sialyl-Tn antigen promotes the secretion of TGF-β, which leads to immunosuppression via the DAP12/Syk pathway (12). Data from The Cancer Genome Atlas (TCGA) showed that Siglec-15 mRNA overexpression had significantly associated with reduced progression-free survival in lung cancer patients, suggesting that Siglec-15 could serve as an important prognostic biomarker (13). Moreover, the expression of Siglec-15 on peritumoral macrophages in primary central nervous system lymphoma correlated with better prognosis (14). Shafi et al. found that Siglec-15 expression in 89% of BC using immunofluorescence, though it was not associated with prognosis (11). Nevertheless, the association between Siglec-15 expression and BC outcomes requires further research.

Although Siglec-15 and PD-L1 share structural similarities, their roles in immune regulation differ, likely driven by distinct signaling pathways (9). Siglec-15 primarily suppresses anti-tumor immunity by regulating the functions of macrophages and dendritic cells, whereas PD-L1 inhibits T cell activation through binding to PD-1. Notably, the expression of Sigle c-15 was mutually exclusive with that of PD-L1: the latter was induced by IFN-γ, whereas Siglec-15 was downregulated. Targeting Siglec-15 could present a new therapeutic approach for patients with low or absent PD-L1 expression, who may not benefit from current PD-1/PD-L1 checkpoint blockade therapies. Since immune checkpoint inhibitors show limited efficacy in tumors with low PD-L1 expression, developing drugs to inhibit Siglec-15 may offer an alternative immunotherapy option (15). Investigating the differential expression of Siglec-15 and PD-L1, along with their effects on immune suppression across various patient subgroups, could help identify those most likely to benefit from tailored immunotherapy approaches.

In this research, we evaluated the expression of PD-L1 and Siglec-15 using immunohistochemical staining. We analyzed the association of PD-L1 and Siglec-15 expression with clinicopathological features and prognostic parameters in BC.

## Materials and methods

### Patient cohort and follow-up

This study comprised of 69 BC patients (age, 43-92 years), who underwent radically cystectomy at Nanjing Drum Tower Hospital (Nanjing, China) from 2015 to 2017. Tissue samples were formalin fixed and paraffin embedded (FFPE). The tumor tissues were pathologically confirmed to be invasive high-grade urothelial carcinoma of the bladder, including muscle-invasive bladder cancer (MIBC) and non-muscle-invasive bladder cancer (NMIBC). The following demographic and clinicopathological features were collected: age, sex, tumor grade, pathological staging, positive lymph nodes, presence of squamous metaplasia, lymphovascular invasion (LVI) and perineural invasion (PNI), and follow-up data. The experiment was ethically approved by the Ethics Committee of Nanjing Drum Tower Hospital (No. 2021-452-01).

### Immunohistochemical staining and assessment for Siglec-15 and PD-L1 expression

Tissue samples were FFPE and sectioned at a thickness of 4 µm. For staining, the deparaffinized sections were immersed in xylene and hydrated with decreasing concentrations of ethanol. Slides were boiled in sodium citrate antigen retrieval buffer (10 mM, pH 6.0) for 20 minutes. After cooling to room temperature, slides were immersed in a methanol solution containing 3% H_2_O_2_ to block endogenous peroxidase activity for 25 minutes. The sections were then incubated with the rabbit anti-Siglec-15 polyclonal antibody (PA5-72765; Invitrogen, Carlsbad, CA, USA; 1:500 dilution) at 4°C overnight. Finally, sections were developed with 3.3′-diaminobenzidine (Envision system 2-Solution DAB Kit), counterstained with Carazzi’s hematoxylin, dehydrated in alcohol, cleared with xylene, and mounted.

**Figure 1:**
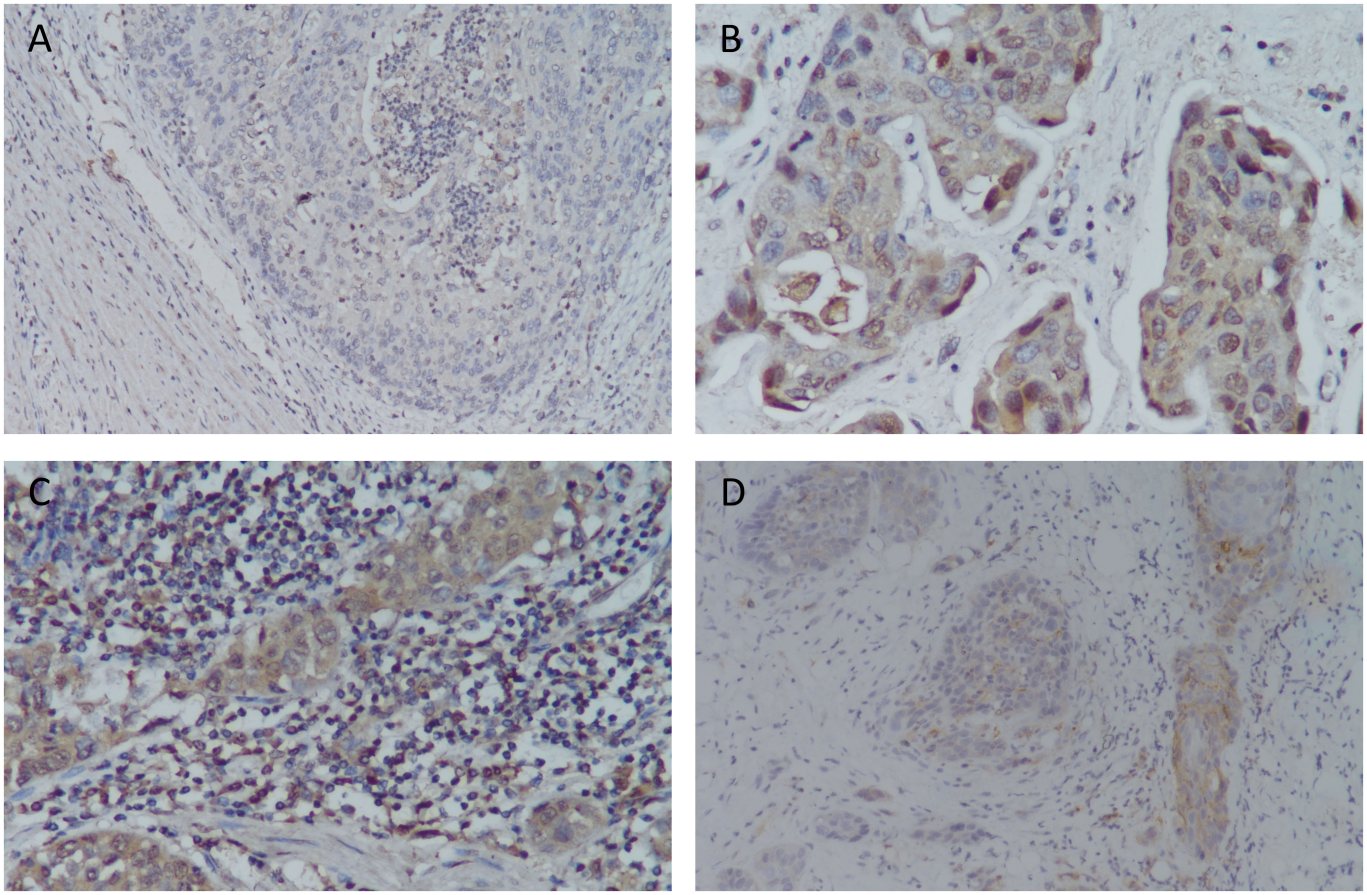
Siglec-15 and PD-L1 expression by immunohistochemistry in bladder cancer tissue samples. **(A)** Siglec-15 expression in intratumoral mononuclear infiltrating cells in bladder cancer (IRS: 1), 200x; **(B)** Siglec-15 cytoplasmic expression in tumor cells (IRS: 12), 400x; **(C)** Siglec-15 expression in stromal mononuclear infiltrating cells (IRS: 9) and tumor cells (IRS: 8), 400x; **(D)** PD-L1 expression in tumor cells and infiltration of immune cells (CPS: 30), 200x.

The positive control reactions were human kidney and prostate cancer tissue. Negative control reactions were performed by omitting the primary antibody from the dilution buffer. Immunohistochemical evaluations of Siglec-15 expression were independently performed by two pathologists using Olympus BX41 microscopes. In BC, Siglec-15 protein expression was detected both in tumor cells and in cells from the tumor microenvironment, including mononuclear immune cells (**Figure 1**). Siglec-15 expression on tumor cells and tumor-infiltrating stromal/immune cells were assessed by IHC. For positive staining in tumor and stroma, the most intensively stained region was initially selected with a low-power magnification (×100). The percentage of positively stained cells was then calculated from the observation of 5 random sections at a higher magnification (×200). A total of 100 tumor cells were counted in each section, and the number and intensity classification of the positive-stained cells was determined. The immunoreactive score (IRS) system was used. The staining intensity classification was as follows: 0, unstained; 1, light yellow; 2, brownish yellow; and 3, tan. The percentage score for positive cells was classified as follows: 0 is negative; 1, 10% positive cells; 2, 11-50% positive cells; 3, 51-80% positive cells; and 4, more than 80% positive cells. IRS = SI (staining intensity) x PP (percentage of positive cells). Based on IRS, the staining was categorized as negative (IRS, 0) and positive (IRS, 1-12). The association between the expression of Siglec-15 and pathological parameters of patients was analyzed.

PD-L1 expression was assessed utilizing the pharmDx immunohistochemistry assay (PD-L1 IHC 22C3) and recorded according to the combined positivity score (CPS) formula. The CPS formula calculates the percentage of PD-L1-positive cells (tumor cells, lymphocytes, macrophages) relative to the total number of tumor cells. A CPS score of ≥10 was deemed indicative of positivity. This methodology adhered to Food and Drug Administration (FDA) guidelines for PD-L1 testing in urothelial carcinoma (16).

### Statistical analysis

The relationship between Siglec-15, PDL1 expression and clinicopathologic parameters was analyzed by the χ2 test and Fisher’s exact test. Spearman’s rank correlation was used to analyze the correlation between Siglec-15 and PD-L1 expression. Overall survival (OS) and cancer specific survival (CSS) were estimated by the Kaplan-Meier method with the log-rank test. Survival curves were generated using the R package survfit function. Cox hazard proportion model was performed for multivariate analysis. Two-side *P* < 0.05 was regarded statistically significant difference. The statistical software SPSS v21.0 (IBM Corporation, Armonk, NY, USA) and R software v4.1.1 were used.

## Results

### The expression of Siglec-15 and PD-L1

**Figure 2:**
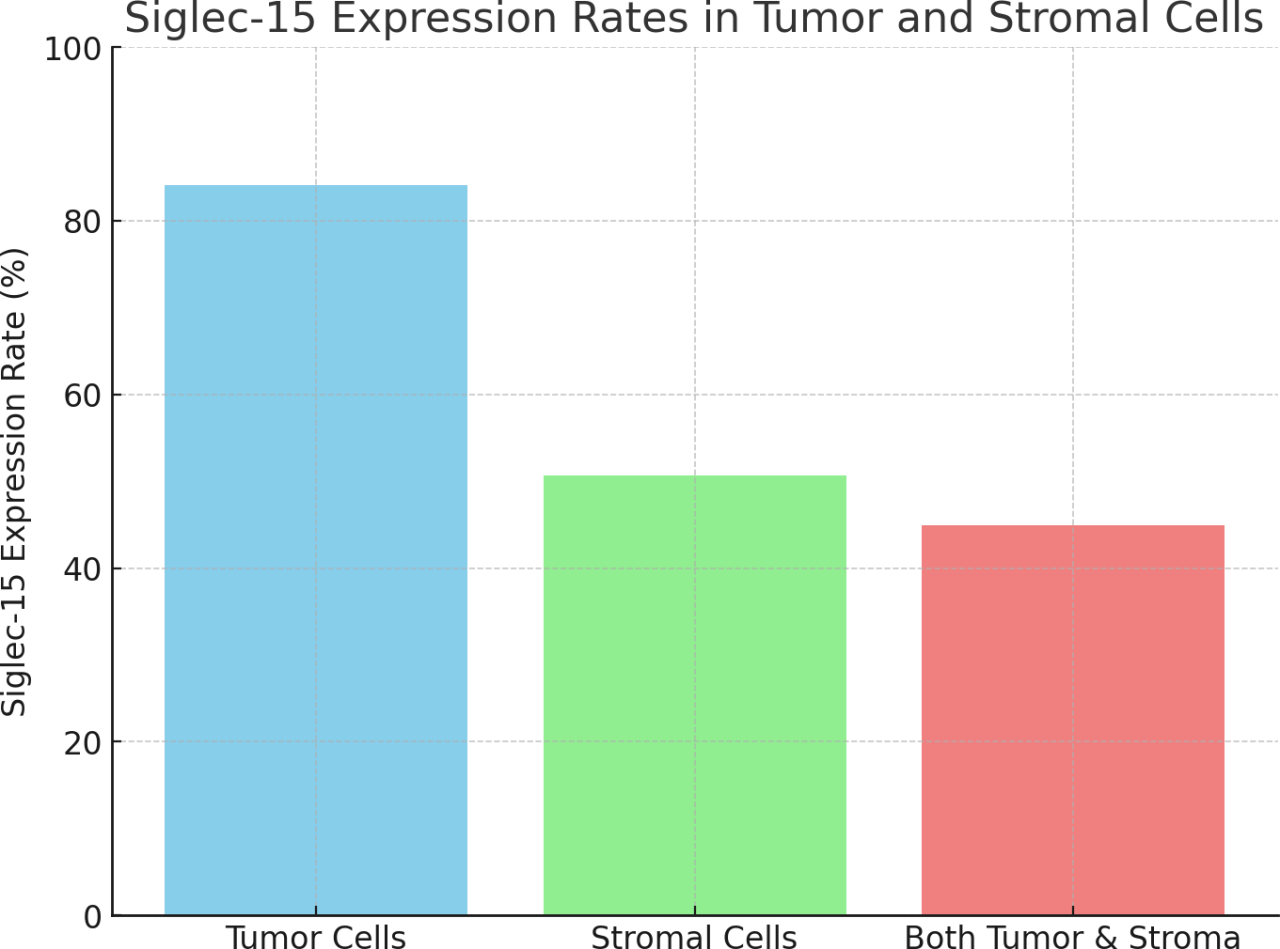
The bar chart representing the Siglec-15 expression rates: 84.1% in tumor cells, 50.7% in stromal cells, and 44.9% in both tumor and stroma.

We enrolled 69 patients (ages 43-92 years) with primary urothelial carcinoma of the bladder. Siglec-15 expression rates were observed as follows: 84.1% (58/69) in tumor cells, 50.7% (35/69) in stromal cells, and 44.9% (31/69) in both tumor and stroma (**Figure 2**). Among the tumor cells, 33 (56.9%) showed cytoplasmic Siglec-15 expression, while 25 (43.1%) displayed nuclear Siglec-15 expression (**Figures 1A-C**). Regarding the intensity of Siglec-15 expression in tumor cells, 28 cases (47.5%) were mildly positive (IRS 1–3), 26 cases (44.8%) were moderately positive (IRS 4–8), and 4 cases (6.9%) were strongly positive (IRS 9–12). Additionally, 25 cases (71.4%) exhibited mildly positive stromal cell expression, while 10 cases (28.6%) showed moderate expression, with no instances of strong expression observed. There was no significant correlation between tumoral and stromal Siglec-15 expression (R = 0.125*, P* = 0.306). PD-L1 status was available for all 69 bladder cancer samples we examined. The expression rate of PD-L1 in BC samples was 52.2% (36/69), with a positivity rate (CPS≥10) of 17.4% (12/69) (**Figure 1D**). Although an inverse correlation with PD-L1 and Siglec-15 was noted, statistical significance was not achieved (R = -0.220, *P* = 0.072).

### Relationship between PD-L1 and Siglec-15 expression and clinicopathological characteristics

The clinicopathological features of the patients, categorized by Siglec-15 and PD-L1 status, are summarized in **Table 1**. Regarding the Siglec15 and its relationship with clinicopathological parameters, tumoral expression was significantly higher in male patients compared to female patients (*P* = 0.038). However, no significant associations were found between tumoral Siglec-15 expression and other clinicopathological features, including age, T stage, N stage, LVI, PNI, squamous metaplasia, and survival status. Additionally, we also compared the differences in Siglec-15 expression intensity with clinicopathological parameters (**Supplementary Table S2**). The intensity of tumoral Siglec-15 expression was associated with the patient’s gender (*P* = 0.011). and lymph node metastasis (*P* = 0.027). Interestingly, the status and intensity of stromal Siglec-15 expression were associated with BC survival outcomes, age, and T staging. Low-stage carcinoma exhibited higher stromal Siglec-15 expression compared to high-stage carcinoma (*P* = 0.010). In other words, the stromal expression level of Siglec-15 was higher in NMIBC compared to MIBC (*P* = 0.014). PD-L1 expression correlated with total pathological stage (*P* = 0.008) and PNI (*P* = 0.048), but not with other parameters.

**Table 1:** Clinicopathological features stratified by Siglec-15 and PD-L1 status.

Variable	Total, N = 69 (%)	Tumoral Siglec-15			Stromal Siglec-15			PD-L1		
		Positive, N =58 (%)	Negative, N = 11(%)	*P* Value	Positive, N = 35 (%)	Negative, N = 34 (%)	*P* Value	*CPS*		*P* Value
								*<10*	*≥10*	
**Age(years)**				0.627			**0.040**			0.638
≥65	33(47.8)	27(46.6)	6(54.5)		21(60.0)	12(35.30		28(49.1)	5(42.7)	
<65	36(52.2)	31(53.4)	5(45.5)		14(40.0)	22(64.7)		29(50.9)	7(58.3)	
**Sex**				**0.038**			0.348			0.056^a^
Male	54(78.3)	48(82.8)	6(54.5)		29(73.5)	25(82.9)		42(73.7)	12(100.0)	
Female	15(21.7)	10(17.2)	5(45.5)		6(26.5)	9(17.1)		15(26.3)	0	
**T stage**				0.356			**0.010**			**0.008**
T1	17(24.6)	14(24.6)	3(27.3)		13(24.6)	4(11.8)		15(26.3)	2(16.7)	
T2	40(58.0)	34(58.0)	6(54.5)		20(57.1)	20(58.8)		34(59.2)	6(50.0)	
T3	6(8.7)	6(8.7)	0(0)		2(5.7)	4(11.8)		6(10.5)	0(0)	
T4	6(8.7)	6(8.7)	2(18.2)		0(0)	6(17.6)		2(3.5)	4(33.3)	
**N stage**				0.422			0.326			0.548
N0	56(81.2)	48(82.8)	8(72.7)		30(85.7)	26(76.5)		47(82.5)	9(75.0)	
N+	13(18.8)	10(17.2)	3(27.3)		5(14.3)	8(23.5)		10(17.5)	3(25.0)	
**LVI**				0.188			0.113			
No	43(62.3)	34(58.6)	9(81.8)		25(71.4)	18(52.9)		36(63.2)	7(58.3)	
Yes	26(37.7)	24(41.4)	2(18.2)		10(28.6)	16(47.1)		21(36.8)	5(41.7)	
**PNI**				0.490 ^a^			0.095			0.048^a^
No	49(71.0)	20(69.0)	9(81.8)		28(80.0)	21(61.8)		39(68.4)	10(83.3)	
Yes	20(29.0)	18(31.0)	2(18.2)		7(20.0)	13(38.2)		18(31.6)	2(16.7)	
**Squamous metaplasia**				0.364 ^a^			0.703			0.390
No	58(84.1)	50(86.2)	8(72.7)		30(85.7)	28(82.4)		49(86.0)	9(75.0)	
Yes	11(15.9)	8(13.8)	3(27.3)		5(14.3)	6(17.6)		8(14.0)	3(25.0)	
**Status**				0.516 ^a^			**0.010**			0.333
Alive	43(62.3)	35(60.3)	8(72.7)		27(77.1)	16(47.1)		37(64.9)	6(50.0)	
Death	26(37.7)	23(39.7)	3(27.3)		8(22.9)	18(52.9)		20(35.1)	6(50.0)	

a Fisher-exact test. Bold value indicates a significant difference.

LVI, Lymphovascular invasion; PNI, Perineural invasion.

### Association of PD-L1 and Siglec-15 expression with clinical outcomes

**Figure 3:**
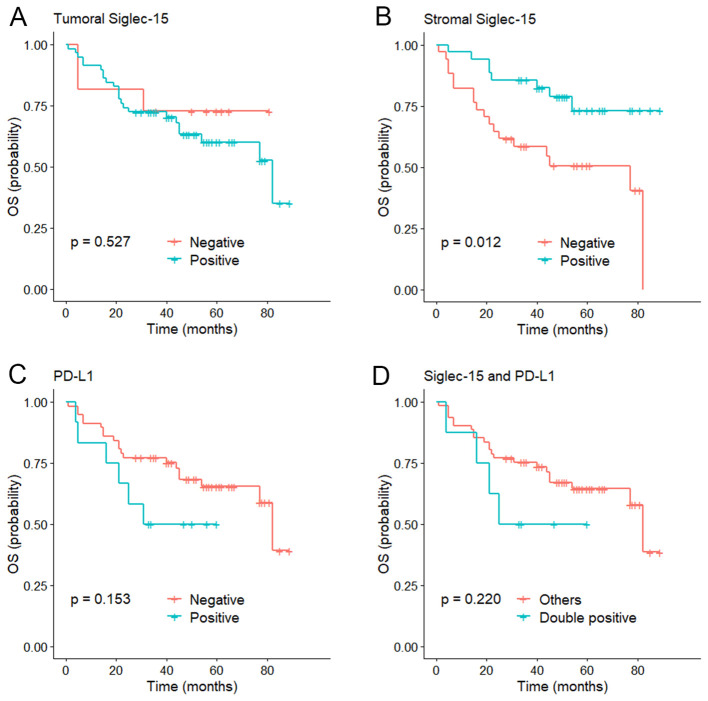
Kaplan–Meier curve of overall survival (OS). **(A)** According to the tumoral Siglec-15 expression; **(B)** According to the stromal Siglec-15 expression. **(C)** According to PD-L1 expression. **(D)** According to both Siglec-15 and PD-L1 positive.

**Figure 4:**
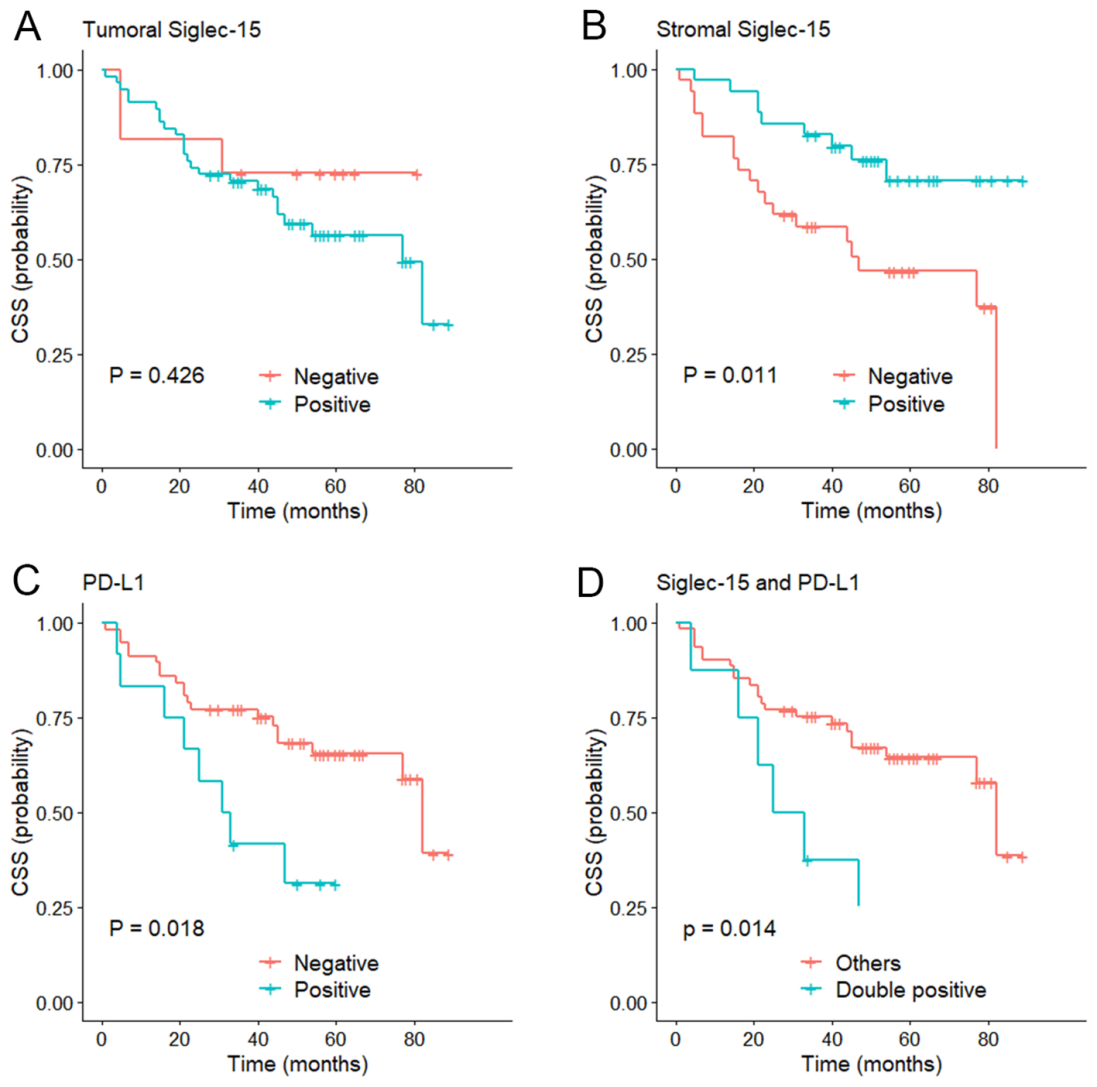
Kaplan–Meier curve of cancer specific survival (CSS). **(A)** According to the tumoral Siglec-15 expression; **(B)** According to the stromal Siglec-15 expression. **(C)** According to PD-L1 expression. **(D)** According to both Siglec-15 and PD-L1 positive.

The median follow-up of the series was 56 months (range 1-89 months). Kaplan-Meier curves showed that stromal Siglec-15 positivity was significantly linked to better OS (*P* = 0.012, **Figure 3B**) and CSS (*P* = 0.011, **Figure 4B**). In contrast, tumoral Siglec-15 expression did not display a significant correlation with either OS (*P* = 0.527, **Figure 3A**) or CSS (*P* = 0.426, **Figure 4A**). PD-L1 overexpression was significantly associated with worse CSS (*P* = 0.018, **Figure 4C**), but not with OS (*P* = 0.153, **Figure 3C**). Co-expression of Siglec-15 and PD-L1 also did not correlate with OS (*P* = 0.220, **Figure 3D**), but was significantly associated with worse CSS (*P* = 0.014, **Figure 4D**). Univariate Cox regression analysis showed that age, T stage, LVI, PNI, stromal Siglec-15 status were associated with OS (**Table 2**). However, multivariate analysis revealed that stromal Siglec-15 expression is not independently associated with prognosis (*P* = 0.236).

**Table 2:** Univariate and multivariate analyses of clinicopathological and Siglec-15 status for overall survival.

Variables	Univariate analysis		Multivariate analysis	
	HR (95% CI)	*P* value	HR (95% CI)	*P* value
**Age(years)**	2.839(1.190-6.774)	**0.019**	2.173(0.874-5.402)	0.095
**Sex**	1.060(0.397-3.829)	0.908		
**T stage**	2.031(1.369-3.013)	**0.001**	1.268(0.785-2.047)	0.331
**N stage**	2.020(0.934-4.895)	0.119		
**LVI**	5.302(2.247-12.510)	**0.001**	4.247(1.576-11.446)	**0.004**
**PNI**	2.511(1.159-5.441)	**0.020**	0.912(0.378-2.203)	0.838
**Squamous metaplasia**	2.119(0.839-5.357)	0.113		
**PD-L1 CPS**	1.935(0.765-4.893)	0.153		
**Tumoral Siglec-15**	1.476(0.441-4.939)	0.527		
**Stromal Siglec-15**	0.342(0.148-0.789)	**0.012**	0.569(0.224-1.447)	0.236

HR, hazard ratio; CI, confidence interval; LVI, Lymphovascular invasion; PNI, Perineural invasion.

Bold value indicates a significant difference.

## Discussion

The association between Siglec-15 expression and outcomes in BC patients was investigated through immunohistochemical staining of 69 BC specimens. We found the 84.1% of BC specimens expressed Siglec-15 in bladder tumor cells. High Siglec-15 staining scores in the stromal immune cells of BC was associated with lower T staging and a favorable prognosis, even though it was not identified as an independent prognostic factor.

Siglecs are sialic-acid-binding immunoglobulin-like lectins predominantly expressed by cells of the immune system. Siglec-15 is notably upregulated in various human cancers and functions as a key immunosuppressive factor (9). Siglec-15 is primarily expressed on the cell membrane. As a transmembrane protein, Siglec-15 is typically localized on the surface of the cell membrane, especially in tumor cells and tumor-associated immune cells such as macrophages and dendritic cells. Its membrane expression facilitates interactions between cells, particularly during immune evasion, where it helps suppress the immune system’s attack on tumor cells. However, we observed that Siglec-15 is expressed in both the cytoplasm and on the cell membrane. The cytoplasmic staining may reflect intracellular stores of Siglec-15, which may be involved in its trafficking to the cell surface or reflect newly synthesized protein in the secretory pathway. The specific significance and mechanisms of Siglec-15 expression in both the cytoplasm and cell membrane require further research.

We found a significant association between PD-L1 expression and pathological T stage, as most tumors that expressed PD-L1 were of high stage, similar to the results of other studies (17, 18). Interestingly, we also observed that Siglec-15 was associated with high-stage tumors. Our data showed an inverse correlation between Siglec-15 expression and PD-L1, but statistical significance was not achieved. A study on multiple types of solid tumors has revealed that the expression of Siglec15 exhibits significant heterogeneity and is negatively correlated with PD-L1 (19). The expression of Sigle c-15 was mutually exclusive with that of PD-L1: the latter was induced by IFN-γ, whereas Siglec-15 was downregulated. Targeting Siglec-15 could present a new therapeutic approach for patients with low or absent PD-L1 expression, who may not benefit from current PD-1/PD-L1 checkpoint blockade therapies (15). Analysis of TCGA databases revealed widespread expression of Siglec-15 mRNA in BC, lung cancer, renal cell carcinoma, colon cancer and other malignancies (10, 13). Immunofluorescence staining analysis in non-small cell lung cancer tissues indicated upregulation of Siglec-15. Further studies have shown higher Siglec-15 expression levels in lung adenocarcinoma compared to squamous cell carcinoma (20). In pancreatic ductal adenocarcinoma, Siglec-15 was expressed in 18.6% of cases and associated with higher tumor grade (19). Siglec-15 functions as a receptor that can directly bind to T cells, although it was not as well-known as immune receptors of B7 family. Previous research suggested that patients who do not respond effectively with PD-1/PD-L1 monoclonal antibody might benefit from Siglec-15 monoclonal antibody therapy (21, 22). Promising results from a phase I clinical trial using the Monoclonal Antibody Targeting Siglec-15 (NC 318) support this potential therapeutic approach (23).

Previous studies have yielded mixed findings regarding the prognostic implications of Siglec-15 expression. The pan-cancer analyses indicated that Siglec-15 serves as a pan-cancerous prognostic biomarker, with higher levels of correlating generally with poorer outcomes (13). However, specific studies have reported varying associations depending on the cancer type. For instance, Chen et al. (19) observed that Siglec-15 overexpression was linked to better outcomes in patients with pancreatic ductal adenocarcinoma. Similarly, in primary central nervous system diffuse large B-cell lymphoma, Siglec-15 expression in peri-tumor macrophages was associated with improved prognosis (14). Conversely, high Siglec-15 expression has been predictive of dismal prognosis in osteosarcoma and nasopharyngeal carcinoma (24, 25). Several studies across different cancer types, including lung, breast, head and neck, gastric, and BC, have indicated that Siglec-15 expression was not consistently associated with prognosis (11, 20, 26). In our present study, we evaluated Siglec-15 expression in both stroma and tumor cells of BC. We found that Siglec-15 expression levels in tumor cells were not associated with the prognosis. Interestingly, high stromal Siglec-15 expression was associated with a favorable prognosis, although it was not identified as an independent prognostic factor for BC.

Further studies should focus on the immune evasion mechanism of Siglec-15 in various carcinomas. Wang et al. discovered that Siglec-15 expression was induced by macrophage colony-stimulating factor and surperssed by IFN-γ, thereby inhibiting antigen-specific T cell response (9). Liu et al. demonstrated that Siglec-15 promotes the migration of hepatocellular carcinoma cells by regulating the stability of CD44 protein (27). He et al. revealed that anti-siglec-15 monoclonal antibodies can effectively block Siglec-15-mediated T cell inhibition and inhibit tumor growth (28). Additionally, Gao et al. confirmed through cell experiments that Siglec-15 significantly inhibited the proliferation, migration, and invasion of BC cells (29). Li et al. revealed that the BACH1-IT2-miR-4786-Siglec-15 axis plays a crucial role in immune evasion in BC by stabilizing Siglec-15 and contributing to a local immune suppressive microenvironment (30). Therefore, Siglec-15 may serve bot only as a prognostic biomarker but also as a potential therapeutic target to enhance cancer immunotherapy for BC.

The limitations of this study are as follows. Firstly, it is a retrospective case-control study with a limited sample size. Secondly, this study lacks biological experiments to elucidate the role of Siglec-15 in BC both *in vivo* or *in vitro*. Moreover, at the initiation of this study, no commercial monoclonal antibody against Siglec-15 was available. Additionally, there is currently no standard criteria for positive immunohistochemical staining of Siglec-15. More comprehensive, extensive and in-depth analyses are needed in the future.

In conclusion, our study indicates that Siglec-15 is broadly expressed in BC, including tumor cells and stromal immune cells. Siglec-15 overexpression in the stroma of BC was associated with lower T staging and improved prognosis, but not functioned as an independent prognostic factor. These findings provide important insights into the prognosis and survival outcomes for BC patients, potentially guiding patient selection in future clinical trials.

## Data Availability

The raw data supporting the conclusions of this article will be made available by the authors, without undue reservation.

## References

[1] Siegel RL Miller KD Jemal A. Cancer statistics, 2016. CA Cancer J Clin. (2016) 66:7–30. doi: 10.3322/caac.21332 26742998

[2] van Hoogstraten LMC Vrieling A van der Heijden AG Kogevinas M Richters A Kiemeney LA. Global trends in the epidemiology of bladder cancer: challenges for public health and clinical practice. Nat Rev Clin Oncol. (2023) 20:287–304. doi: 10.1038/s41571-023-00744-3 36914746

[3] Pang C Guan Y Li H Chen W Zhu G. Urologic cancer in China. Japan J Clin Oncol. (2016) 46:497–501. doi: 10.1093/jjco/hyw034 27049022

[4] Patel VG Oh WK Galsky MD. Treatment of muscle-invasive and advanced bladder cancer in 2020. CA Cancer J Clin. (2020) 70:404–23. doi: 10.3322/caac.21631 32767764

[5] Rose TL Deal AM Nielsen ME Smith AB Milowsky MI. Sex disparities in use of chemotherapy and survival in patients with advanced bladder cancer. Cancer. (2016) 122:2012–20. doi: 10.1002/cncr.v122.13 PMC4911298 27224661

[6] Kartolo A Kassouf W Vera-Badillo FE. Adjuvant immune checkpoint inhibition in muscle-invasive bladder cancer: is it ready for prime time? Eur Urol. (2021) 80:679–81. doi: 10.1016/j.eururo.2021.07.019 34366212

[7] Lopez-Beltran A Cimadamore A Blanca A Massari F Vau N Scarpelli M. Immune checkpoint inhibitors for the treatment of bladder cancer. Cancers (Basel). (2021) 13:131. doi: 10.3390/cancers13010131 33401585 PMC7795541

[8] Angata T Tabuchi Y Nakamura K Nakamura M. Siglec-15: an immune system Siglec conserved throughout vertebrate evolution. Glycobiology. (2007) 17:838–46. doi: 10.1093/glycob/cwm049 17483134

[9] Wang J Sun J Liu LN Flies DB Nie X Toki M. Siglec-15 as an immune suppressor and potential target for normalization cancer immunotherapy. Nat Med. (2019) 25:656–66. doi: 10.1038/s41591-019-0374-x PMC7175920 30833750

[10] Li Q-T Huang Z-Z Chen Y-B Yao H-Y Ke Z-H He X-X. Integrative analysis of siglec-15 mRNA in human cancers based on data mining. J Cancer. (2020) 11:2453–64. doi: 10.7150/jca.38747 PMC7066007 32201516

[11] Shafi S Aung TN Xirou V Gavrielatou N Vathiotis IA Fernandez A. Quantitative assessment of Siglec-15 expression in lung, breast, head, and neck squamous cell carcinoma and bladder cancer. Lab Invest. (2022) 102 (10):1143–9. doi: 10.1038/s41374-022-00796-6 35581307 PMC10211373

[12] Rodrigues Mantuano N Natoli M Zippelius A Läubli H. Tumor-associated carbohydrates and immunomodulatory lectins as targets for cancer immunotherapy. J Immunother Cancer. (2020) 8:e001222. doi: 10.1136/jitc-2020-001222 33020245 PMC7537339

[13] Li B Zhang B Wang X Zeng Z Huang Z Zhang L. Expression signature, prognosis value, and immune characteristics of Siglec-15 identified by pan-cancer analysis. Oncoimmunology. (2020) 9:1807291–1807291. doi: 10.1080/2162402X.2020.1807291 32939323 PMC7480813

[14] Fudaba H Momii Y Hirakawa T Onishi K Asou D Matsushita W. Sialic acid-binding immunoglobulin-like lectin-15 expression on peritumoral macrophages is a favorable prognostic factor for primary central nervous system lymphoma patients. Sci Rep. (2021) 11:1206–6. doi: 10.1038/s41598-020-79742-9 PMC7806611 33441719

[15] Moreira RS da Silva MM de Melo Vasconcelos CF da Silva TD Cordeiro GG Mattos-Jr LAR. Siglec 15 as a biomarker or a druggable molecule for non-small cell lung cancer. J Cancer Res Clin Oncol. (2023) 149:17651–61. doi: 10.1007/s00432-023-05437-z PMC11798167 37843557

[16] Necchi A Anichini A Raggi D Briganti A Massa S Lucianò R. Pembrolizumab as neoadjuvant therapy before radical cystectomy in patients with muscle-invasive urothelial bladder carcinoma (PURE-01): an open-label, single-arm, phase II study. J Clin Oncol. (2018) 36:3353–60. doi: 10.1200/JCO.18.01148 30343614

[17] Seranio N Malkowicz S Aguarin L Dorsey J Christodouleas J Kao GJJTS. Predicting bladder cancer responses to PD-L1 inhibitors? A Case Rep overview busy clinic. (2020) 6:1–5.

[18] Al Nabhani S Al Harthy A Al Riyami M Al Sinawi S Al Rashdi A Al Husseni S. Programmed death-ligand 1 (PD-L1) expression in bladder cancer and its correlation with tumor grade, stage, and outcome. Oman Med J. (2022) 37:e441. doi: 10.5001/omj.2022.96 36458243 PMC9631119

[19] Chen X Mo S Zhang Y Ma H Lu Z Yu S. Analysis of a novel immune checkpoint, Siglec-15, in pancreatic ductal adenocarcinoma. J Pathol Clin Res. (2022) 8:268–78. doi: 10.1002/cjp2.v8.3 PMC8977273 35083884

[20] Hao JQ Nong JY Zhao D Li HY Su D Zhou LJ. The significance of Siglec-15 expression in resectable non-small cell lung cancer. Neoplasma. (2020) 67:1214–22. doi: 10.4149/neo_2020_200220N161 32749846

[21] Ren X. Immunosuppressive checkpoint Siglec-15: a vital new piece of the cancer immunotherapy jigsaw puzzle. Cancer Biol Med. (2019) 16:205–10. doi: 10.20892/j.issn.2095-3941.2018.0141 PMC6713637 31516742

[22] Cao G Xiao Z Yin Z. Normalization cancer immunotherapy: blocking Siglec-15! Signal Transduct Target Ther. (2019) 4:10. doi: 10.1038/s41392-019-0045-x 31016034 PMC6473001

[23] Chen R Manochakian R James L Azzouqa AG Shi H Zhang Y. Emerging therapeutic agents for advanced non-small cell lung cancer. J Hematol Oncol. (2020) 13:58. doi: 10.1186/s13045-020-00881-7 32448366 PMC7245927

[24] Fan M-K Zhang G-C Chen W Qi L-L Xie M-F Zhang Y-Y. Siglec-15 promotes tumor progression in osteosarcoma via DUSP1/MAPK pathway. Front Oncol. (2021) 11:710689–9. doi: 10.3389/fonc.2021.710689 PMC8322944 34336699

[25] Zhao J Yang H Hu H Liu C Wei M Zhao Y. Prognostic value of PD-L1 and Siglec-15 expression in patients with nasopharyngeal carcinoma. Sci Rep. (2022) 12:10401–1. doi: 10.1038/s41598-022-13997-2 PMC9213533 35729260

[26] Quirino MWL Pereira MC Deodato de Souza M Pitta I Da Silva Filho AF Albuquerque M. Immunopositivity for Siglec-15 in gastric cancer and its association with clinical and pathological parameters. Eur J histochem: EJH. (2021) 65:3174. doi: 10.4081/ejh.2021.3174 33666065 PMC7967265

[27] Liu W Ji Z Wu B Huang S Chen Q Chen X. Siglec-15 promotes the migration of liver cancer cells by repressing lysosomal degradation of CD44. FEBS Lett. (2021) 595:2290–302. doi: 10.1002/1873-3468.14169 34328657

[28] He F Wang N Li J He L Yang Z Lu J. High affinity monoclonal antibody targeting Siglec-15 for cancer immunotherapy. J Clin Trans Res. (2021) 7:739–49. PMC8710358 34988324

[29] Gao H-Y Liu G-J Qiu Y-R. Siglec-15 regulates cell proliferation, migration and invasion in bladder cancer. Asian J Surg. (2024) 47:1892–4. doi: 10.1016/j.asjsur.2023.12.140 38169162

[30] Li X Liang Z Pan J Zhang M Liu J Hu R. Identification of BACH1-IT2-miR-4786-Siglec-15 immune suppressive axis in bladder cancer. BMC Cancer. (2024) 24:328. doi: 10.1186/s12885-024-12061-8 38468240 PMC10926634

