# Supplementary material for: Stromal immune cells expression of Siglec-15 is associated with lower T stage and better prognosis of urinary bladder cancer
Source: Front Oncol. 2024 Dec 18;14:1437006. doi: 10.3389/fonc.2024.1437006 (PMC11688221; doi:10.3389/fonc.2024.1437006)
Supplement: Supplementary file 1 [file Table1.docx]

**Table S1.** Siglec-15 expression level in various cancers based on IHC, TCGA data.

| **Cancer Type** | **Siglec-15 Expression (IHC)** | **Expression Based on TCGA** |
| --- | --- | --- |
| Lung Cancer | High | High |
| Head and neck cancer | High | Moderate |
| Breast Cancer | Low | Low |
| Pancreatic Cancer | Moderate | Moderate |
| Bladder cancer | High | High |
| Clear Cell Renal Cell Carcinoma | Low | Low |
| Melanoma | Moderate | High |
| Colorectal Cancer | High | High |

**Table S2.** The relationship between IRS of Siglec-15 and clinicopathological parameters.

| **Variable** | **Total, N = 69** | **Tumoral Siglec-15** | | | | | **Stromal Siglec-15** | | | |
| --- | --- | --- | --- | --- | --- | --- | --- | --- | --- | --- |
|  |  | **Negative**  **IRS 0,**  **N = 11** | **Mild**  **IRS 1-3,**  **N = 28** | **Moderate**  **IRS 4-8,**  **N= 26** | **Strong**  **IRS 9-12,**  **N = 4** | ***P* Value** | **Negative**  **IRS 0,**  **N = 34** | **Mild**  **IRS 1-3,**  **N = 25** | **Moderate**  **IRS 4-8,**  **N= 10** | ***P* Value** |
| **Age（years）** | |  |  |  |  | 0.440 |  |  |  | **0.045** |
| ≥65 | 33(47.8) | 5(45.5) | 17(60.7) | 11(42.3) | 3(75.0) |  | 22(64.7) | 8(32.0) | 6(60.0) |  |
| ＜65 | 36(52.2) | 6(54.5) | 11(39.3) | 15(57.7) | 1(25.0) |  | 12(35.3) | 17(68.0) | 4(40.0) |  |
| **Sex** |  |  |  |  |  | **0.011** |  |  |  | 0.576 |
| Male | 54(78.3） | 6(54.5) | 20(71.4) | 25(96.2) | 3(75.0) |  | 25(73.5) | 20(80.0) | 9(90.0) |  |
| Female | 15(21.7) | 5(45.5) | 8(28.6) | 1(3.8) | 1(25.0) |  | 9(26.5) | 5(20.0) | 1(10.0) |  |
| **T stage** |  |  |  |  |  | 0.899 |  |  |  | **0.030** |
| T1 | 17(24.6) | 3(27.3) | 5(17.9) | 8(30.8) | 1(25) |  | 4(11.8) | 11(44.0) | 2(20.0) |  |
| T2 | 40(58.0) | 6(54.5) | 18(64.3) | 13(50.0) | 3(75.0) |  | 20(58.8) | 13(52.0) | 7(70.0) |  |
| T3 | 6(8.7) | 0(0) | 3(10.7) | 3(11.5) | 0(0) |  | 4(11.8) | 1(4.0) | 1(10.0) |  |
| T4 | 6(8.7) | 2(18.2) | 2(2.9) | 2(2.9) | 0(0) |  | 6(17.6) | 0(0) | 0 |  |
| **N stage** |  |  |  |  |  | **0.027** |  |  |  | 0.282 |
| N0 | 56(81.2) | 8(72.7) | 27((96.4) | 18(69.2) | 3(75.0) |  | 26(76.5 | 10(80.0) | 10(100) |  |
| N+ | 13(18.8) | 3(27.3) | 1(3.6) | 8(30.8) | 1(25.0) |  | 8(23.5 | 5(20.0) | 0(0) |  |
| **LVI** |  |  |  |  |  | 0.294 |  |  |  | 0.198 |
| No | 43(62.3) | 9(81.8) | 18(64.3) | 13(50.0) | 3(75.0) |  | 18(52.9) | 19(76.0) | 6(60.0) |  |
| Yes | 26(37.7) | 2(18.2) | 10(35.7) | 13(50.0) | 1(25.0) |  | 16(47.1) | 6(24.0) | 4(40.0) |  |
| **PNI** |  |  |  |  |  | 0.659 |  |  |  | 0.192 |
| No | 49(71.0) | 9(81.8) | 19(67.9) | 19(73.1) | 2(50.0) |  | 21(61.8) | 19(76.0) | 9(90.0) |  |
| Yes | 20(29.0) | 2(18.2) | 9(32.1) | 7(26.9) | 2(50.0) |  | 13(38.2) | 6(24.0) | 1(10.0) |  |
| **Squamous metaplasia** | | |  |  |  | 0.088 |  |  |  | 0.031 |
| No | 58(84.1) | 8(72.7) | 22(78.6) | 25(96.2) | 3(75.0) |  | 28(82.4) | 24(96.0) | 6(60.0) |  |
| Yes | 11(15.9) | 3(27.3) | 6(21.4) | 1(3.8) | 1(25.0) |  | 6(17.6) | 1(4.0) | 4(40.0) |  |
| **Status** |  |  |  |  |  | 0.775 |  |  |  | **0.030** |
| Alive | 43(62.3) | 8(72.7) | 18(64.3) | 15(57.7) | 2(50.0) |  | 16(47.1) | 20(80.0) | 7(70.0) |  |
| Death | 26(37.7) | 3(27.3) | 10(35.7) | 11(42.3) | 2(50.0) |  | 18(52.9) | 5(20.0) | 3(30.0) |  |

All analyses were performed using Fisher's exact test.
